# Productive Results, Oxidative Stress and Contaminant Markers in European Sea Bass: Conventional vs. Organic Feeding

**DOI:** 10.3390/ani10071226

**Published:** 2020-07-18

**Authors:** Antonio Carminato, Francesco Pascoli, Angela Trocino, Lisa Locatello, Lisa Maccatrozzo, Renato Palazzi, Giuseppe Radaelli, Cristina Ballarin, Martina Bortoletti, Daniela Bertotto

**Affiliations:** 1 Italian Health Authority and Research Organization for Animal Health and Food Safety, Viale dell’Università 14, 35020 Padova, Italy; acarminato@izsvenezie.it (A.C.); fpascoli@izsvenezie.it (F.P.); 2 Department of Comparative Biomedicine and Food Science (BCA), University of Padova, Viale dell’Università 16, 35020 Padova, Italy; angela.trocino@unipd.it (A.T.); lisa.maccatrozzo@unipd.it (L.M.); cristina.ballarin@unipd.it (C.B.); martina.bortoletti@phd.unipd.it (M.B.); daniela.bertotto@unipd.it (D.B.); 3 Department of Biology, University of Padova, Via U. Bassi 58/b, 35121 Padova, Italy; lisa.locatello@unipd.it; 4 Veneto Agricoltura, Innovation and Development Section, Viale dell’Università 14, 35020 Padova, Italy; renato.palazzi@venetoagricoltura.org

**Keywords:** immunohistochemical analysis, RT-PCR, IGFs, GSH, CYP1A, *Dicentrarchus labrax*, organic aquaculture

## Abstract

**Simple Summary:**

Over the years, aquaculture moved to organic production given the rising interest of consumers towards healthy and ecologically friendly food. Among the cultured species, European sea bass (*Dicentrarchus labrax*) is one of the leading farmed fish products in the Mediterranean area and thus one of the most economically important. For these reasons, further investigations on the effects of organic feeding on this species are of primary interest. In the present study, European sea bass were fed two different diets, organic and conventional, and growing performances, oxidative stress, and contaminant markers were determined. Although conventional diet gave the best results in terms of production, groups fed with the organic one also showed a positive growth trend and importantly no negative effects on fish welfare were observed, demonstrating the feasibility of this diet. This work represents an insight into the emerging aquaculture organic production.

**Abstract:**

In the present study European sea bass (*Dicentrarchus labrax*) subjected to two different diets (organic vs. conventional) were evaluated in terms of growing performances, oxidative stress, and contaminant markers. Growing performances were evaluated using biometric measures and condition factor (K), whereas insulin-like growth factor (IGF-I and IGF-II) levels were assessed trough Real-Time PCR analysis. For oxidative stress, immunohistochemical staining for 8-hydroxy-2′-deoxyguanosine (8-OHdG) and 4-hydroxy-2-nonenal (HNE) was performed, whereas total glutathione (GSH) in blood serum was determined by an enzymatic method adapted. Cytochrome P4501A (CYP1A) and melanomacrophage centers (MMCs) were evaluated as contaminant markers trough immunohistochemical and histochemical approaches, respectively. The growing performances showed a positive trend in both groups but a greater productivity in conventional fed fish compared to the organic ones. A significant higher expression of MMCs was observed in organic vs. conventional diet fed fish. Fillet analysis showed a higher MUFA content and a lower PUFAs n-6 content in organically fed sea bass indicating that diets with a content in fatty acids closer to that of wild fish will definitely affect the fatty acid profile of the fish flesh. On the other hand, the diet composition did not seem to affect neither the oxidative stress parameters (GSH, 8-OHdG, HNE) nor the CYP1A expression.

## 1. Introduction

In recent years, the development of organic production responds to a growing consumer request for a high degree of food safety, high nutritional value, sustainable production and eco-environmental attention [1,2]. One of the main topic of the European Commission (EC) organic farming regulation (N.889/2008) [3] is the feed formulations that aim to ensure fish and consumer health and high quality final product with a low environmental impact. Several studies showed the importance of the diet for the optimum growing performances of fishes [4,5,6].

Growth in vertebrates depends on a regulatory network in which the growth hormone (GH)-insulin-like growth factor (IGF)-I axis plays a key role in growth regulation together with insulin, thyroid hormones and sex steroids [7]. While IGF-I mRNA is expressed mainly in liver of adult fish, as in mammals and non-mammalian vertebrates [8,9,10], IGF-II is proved to be ubiquitously expressed, working essentially as a growth factor [11,12,13,14].

Food composition is also considered an important factor in preserving welfare, growth, development, and reproduction of the fish. An important role among all the nutrients is certainly played by the Highly Unsaturated Fatty Acids (HUFAs) [15,16,17], the deficiency of which causes a decreased growth rate and a weakening of immune function. Under farming conditions, the fish are often subjected to unavoidable stressors such as manipulation, size grading, stocking density, fasting, transport, conditions of pre-slaughter and slaughter techniques that could affect health status [18,19,20].

Oxidative stress is considered one of the major upstream components of the signaling cascade involved in many cellular functions [21]. Under conditions of oxidative stress, many effects of cellular dysfunction such as oxidation of proteins, polyunsaturated fatty acids (PUFAs) and DNA are mediated. 4-hydroxy-2-nonenal (HNE) is the most abundant and toxic α,β-unsaturated aldehyde, which originates from the β-cleavage of hydroperoxides from ω-6 PUFAs. It is mainly involved in the inhibition of protein and DNA synthesis and is also considered a potent mutagenic agent [22]. DNA damage may be due to modification of bases, such as the oxidation of deoxyguanosine (dG) to form 8-hydroxy-2′-deoxyguanosine (8-OHdG). If not repaired, this damage can lead to an incorrect pairing between adenine (A) and 8-OHdG, rather than cytosine (C), causing a G:C to T:A transverse mutation [23,24].

High dietary levels of lipids and vitamins likely influence the oxidative status as pointed out by several studies that show a protective effect of such diets [25,26]. In order to prevent oxidation-induced lesions and mortalities, there must be effective antioxidant systems involving compounds such as glutathione (GSH). Total GSH is used as an indicator of oxidative stress due to its action against ROS or molecules such as benzoates and others.

Due to the presence of fish meal and fish oil, commercially marine feed could represent a source of persistent organic pollutants and heavy metals such as mercury, cadmium and arsenic [27,28]. The assessment of the Cytochrome P4501A (CYP1A) in fish represents an environmental biomarker since CYP1A is involved in the biotransformation of a variety of aquatic contaminants such as oil compounds, dioxins, PCBs, and PAHs [29,30,31]. Beyond the liver, which represents the main site of CYP1A expression in fish, the epithelia of organs in direct contact with the environment (gills, intestine and kidney) and the vascular endothelia exhibit a remarkable CYP1A expression as well. Nowadays, melanomacrophage centers’ (MMCs) evaluation is often used as a reliable indicator of pollutant exposure, in particular heavy metals [32,33,34,35] indicating sub-lethal effects [36]. MMCs are groups of pigments containing cells located within the tissues of cold-blooded vertebrates. In fish, MMCs located primarily in the spleen, kidney, and liver [37] can increase after chemical pollutant exposure but also as a result of diseases [38,39] such as chronic inflammatory lesions, ovarian atresia, and changes induced by starvation [40].

The present study was conducted in a commercial farm during the productive season in order to evaluate the potential of organic feeding in European sea bass aquaculture in terms of growth, welfare and product quality. Growth and welfare have been investigated by measure of biometric parameters, growth factor expression, and oxidative stress indicators while fillet composition and contaminant markers have been evaluated to test the effect on the product quality. Tissue microarray technology has been applied to optimize the histological analysis. Although skin coloration might allow to distinguish organically vs. conventionally-produced sea bass [41], this study gives new insights into organic sea bass aquaculture.

## 2. Materials and Methods

### 2.1. Rearing Conditions

**Figure 1:**
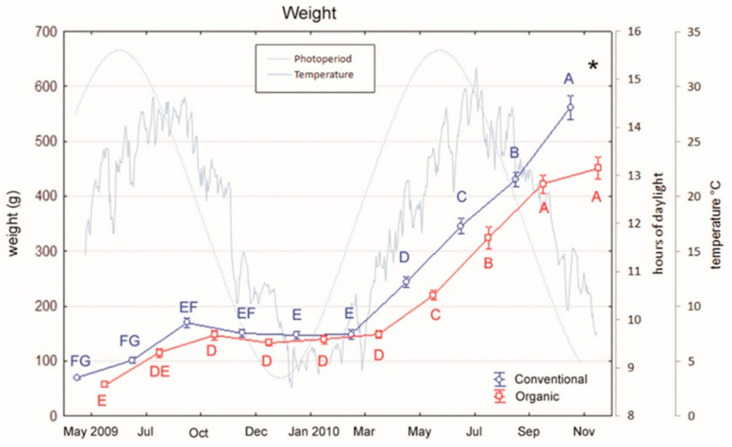
Variations in weight of sea bass over an 18-month period (mean ± SE) reared under conventional and organic aquaculture. Different letters indicate significant differences in samplings within the same feeding system (organic or conventional; *p* < 0.05). Asterisk indicates significant differences between feeding systems (organic vs. conventional) within the same sampling (*p* < 0.05).

European sea bass (initial body weight 62.7 ± 4.0 g) were reared at the “Centro Ittico Valle Bonello” (Porto Tolle, RO, Italy) in a period of the year ranging from April to November into two separated 300 m^3^ outdoor ponds (50 × 6 × 1 m), one for organic and one for conventional feeding trial. All fish had been previously fed a conventional diet and at the start of the trial, fish were moved from the pre-fattening tanks and randomly distributed to the fattening ponds. One of the pond received the conventional diet, the other one was fed with the organic diet. During the trial, fish in the two ponds were fed two times per day by an automatic system and at a rate in the range of 1–2% live weight which was adjusted according to changes in water temperature Figure 1.

In all the ponds, stocking density was the same (initial stocking density: 2 kg/m^3^; 10,000 fish per pond), as all the other parameters (water temperature and photoperiod, both environmental). The dissolved oxygen was kept at normoxic conditions for European sea bass (>7.5 mg/L) as recommended by EFSA (European Food Safety Authority) [40] and ranged from 8 to 12 mg/L in both ponds. Fish of the two groups were fed with different types of diet, one certified organic and one conventional Table 1. Overall mortality at the end of the trial in both the ponds was 15% and final densities were 12 and 14 kg/m^3^ for organic and conventional groups, respectively.

### 2.2. Animal Sampling and Samples Preparation

Fish were monitored for 18 months until they reached the commercial size. Bimonthly, 20 fishes per pond were rapidly netted and immediately after capture, put into iced brackish water and brought to the near laboratory facilities. A big net was used to guarantee a representative sampling and all the sampled animals were subjected to the same procedure that had taken no more than 10 min. Animals were then euthanized with an overdose of anesthetic (MS222, 500 ppm), immediately bled from the caudal vein, and the following organs were collected: liver, spleen, gut, and head kidney. Samples for histology were immediately fixed in 4% paraformaldehyde prepared in phosphate-buffered saline while liver tissue sampled also for molecular analyses, was immediately fixed in trizol and processed as described below. Serum was isolated by allowing the blood to clot overnight at +4 °C and standard centrifugation then conserved at −20 °C until analysis. During each sampling, biometric measures (weight, total and standard lengths) were recorded and Fulton’s condition factor (K) was calculated (K = fish weight/fish total length ^3^) [42]. All animals were treated as requested by EFSA guidelines [43].

### 2.3. Histochemistry

Tissue samples were washed in phosphate-buffered saline (PBS), dehydrated through a graded series of ethanol and embedded in paraffin. Serial sections were cut at a thickness of 4 μm. Haematoxylin and eosin (H & E) staining and Schmorl’s reaction, used to evaluate MMCs for count (see below), were performed as described in Bancroft and Gamble [44].

### 2.4. Tissue Microarray

The array was built using paraffin liver and gut donor blocks of the 200 sampled fishes. Trained technologists using the Beecher TMA instrument (Beecher Instruments, Sun Prairie, WI) removed 1 core of 0.6 mm per donor blocks and transferred it into a recipient block. Cores were arranged in sectors, each containing 4 rows with 10 cores per row following a previously drawn, preset unequivocal plot. Positive and negative control samples were included in each array. Serial 4 μm thick sections were cut.

### 2.5. Immunohistochemistry

Immunohistochemical staining for 8-OHdG, HNE, and CYP1A was performed using the automated immunostainer Benchmark Ultra Ventana as described in Pascoli et al. [34]. Immunoreactive scoring for 8-OHdG and HNE was done by counting positive nuclei in 100 cells. Samples stained with CYP1A were evaluated for the presence and distribution of immunopositivity, and a grade from negative (−) to strong (++) was assigned to the intensity of the reaction.

### 2.6. Melanomacrophage Centers (MMCs) Count

Serial sections of spleen were stained with H & E sequential stain to ascertain structural details, and with Schmorl’s reaction (as described above) to detect the melanomacrophage centers (MMCs). Microscopic quantitative assessment of MMCs was made through a computerized image analyzer system (Olympus CellB, Japan) on sections of spleen since it is the organ which exhibited the highest number of MMCs, as also reported in literature [45]. This quantitative assessment proceeded as follows: (1) Each haul was represented by 3 sections from each spleen. (2) Three fields from each spleen section were analyzed and the amount of MMCs was recorded.

### 2.7. Glutathione (GSH)

Total GSH in blood serum was determined by an enzymatic recycling method adapted for microtitre plate reader [46]. Initially, a standard curve is prepared by diluting GSH Standard stock solution with TF-E (0.1 M phosphate buffer, 0.6 mM EDTA); then in decreasing concentrations of standard solution (SS) thus obtained, are added precise amount of TF-E. Subsequently, two reagents are prepared, the Reaction Solution (RS) and the Reductase. Respectively, for the RS 8.3 mg of NADPH are dissolved in 1 mL of distilled water, to which are then added 0.04 TF-E and 600 μL 5.5‘-ditiobis-2- nitrobenzoic acid (DTNB). For Reductase preparation, 15 μL of the commercial reductase solution (Glutathione Reductase, 205 units/mg protein, Sigma-Aldrich) is diluted with 53.4 μL of ammonium sulphate 3.6 M (Sigma a-4915, MW132.1), then 65 μL of the obtained solution is diluted with 3835 μL of TF-E. A 96 multiwell plate is loaded with 30 μL of TF-E (blank), the standard curve (30 μL of the various points, in descending order) and samples, 15 μL of each in duplicate. The RS is then added and the plate is read at 405 nm (Microplate Photometer Spectracount, Packard Instrument, Meriden, CT, USA) for about ten minutes, until no differences in absorbances were recorded, so the reductase activity is over. At this point, 25 μL of reductase are added to all wells and read on for 20 min. Samples results are compared to standard and expressed in nmol/mL.

### 2.8. Qualitative Reverse Transcription/PCR and Quantitative Real-Time PCR

Qualitative reverse transcription/PCR and quantitative real-time PCR were performed by following the methods detailed in Bertotto et al. [47]. RNAs were extracted from the liver tissue (50 mg sample) of 12 individuals (6 conventional, 6 organic) per sampling period (4 periods: May, December; May and November of the following year) using TRIZOL Reagent (Gibco-BRL, Gaithersburg, MD, USA).

Total RNA (1.5 μg) was retrotranscribed into cDNA. First-strand cDNAs were synthesized by using Superscript II RNase reverse transcriptase protocols (Invitrogen, Life technologies, UK) and a mixture of random hexamers as primer (synthetized by MWG-Biotech, Ebersberg, Germany). The obtained cDNAs were used as templates for PCR expression analysis. We refer the reader to Bertotto et al. [47] for details on IGF-I primer design and efficiency. The primer for IGF-II was designed by using Primer Express 3.0 software (Applied Biosystems) (forward, 5′-AGTGTTGTTTCCGTAGCTGTGA-3′, reverse 5′-ATCCTGAGGGCCAAAAAGTATCG-3′) and its specificity checked by PCRs. Data were normalized to the housekeeping gene β-actin.

Quantification assays to detect the relative expression of IGF-I and IGF-II mRNA were carried out by using the ABI 7500 Real-Time PCR System (Applied Biosystems) as described by Bertotto et al. [47]. Data from SYBR Green I PCR amplicons were collected with ABI 7500 System SDS Software. The ΔΔCt method was used for relative quantification (comparative method) using a calibrator sample as basis for comparative results (see Chemistry Guide, Applied Biosystem, 2003). Dissociation melting curves confirmed the specific amplification of the cDNA target and the absence of nonspecific products.

### 2.9. Proximate Composition and FA Analysis

For proximate composition and fatty acids (FA) analysis, a total of 16 sea bass (8 specimens per rearing system) were collected in January 2011. All fish were slaughtered by immersion in ice slurry and immediately transported to the laboratory in thermally insulated boxes and stored on ice in a refrigerated room (2 °C) for subsequent analysis on the day following collection. Fresh minced fillets were analyzed for FA composition as detailed by Trocino et al. [48].

### 2.10. Statistical Analyses

Statistical analyses were carried out with STATISTICA 9 (StatSoft) and SAS (ver. 9.1) software. All data are reported as mean ± SEM. Data were checked for normality using a Kolmogorov-Smirnov test and in the case of IGF-I and IGF-II expression they were log-transformed to meet the assumption. The effect of feeding condition (organic vs. conventional) and sampling period (10 sampling periods from May to November) on growing performances (weight, length, condition index), IGF-I and IGF-II expression levels, MMCs count and GSH levels were analyzed by means of univariate two-way factorial ANOVAs (GLM). Feeding condition and sampling periods were included in the model as independent fixed factors; growing and oxidative stress parameters as dependent variables. HSD-Tukey’s post-hoc tests were performed when identifying a significant effect. In all analyses a *p* < 0.05 value was considered as statistically significant.

The fillet data collected in the study were analyzed with the GLM procedure of SAS. The diet was used as the experimental factor. Published data [49] were also included for comparison.

## 3. Results

### 3.1. Growth

Fish growth was evaluated using biometric measures and condition factor (K). Since standard and total length were highly correlated (r = 0.99; *p* < 0.001), total length (TL) is considered hereinafter.

**Figure 2:**
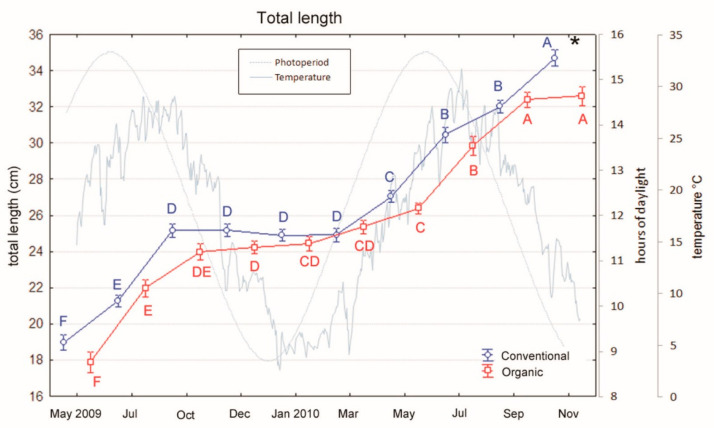
Variations in total length of sea bass over an 18-month period (mean ± SE) reared under conventional and organic aquaculture. Different letters indicate significant differences in samplings within the same feeding system (organic or conventional; (*p* < 0.05). Asterisk indicates significant differences between feeding systems (organic vs. conventional) within the same sampling (*p* < 0.05).

The two-way ANOVA evidenced a significant effect of both the groups and the sampling period on weight (diet: F_1,9_= 16.78, *p* < 0.001; period: F_1,9_ =331.36, *p* < 0.001) and TL (diet: F_1,9_ = 8.99, *p* < 0.001; period: F_1,9_ = 258.01, *p* < 0.001). The interaction between the two factors (diet × period) was also significant for both variables (weight: F_1,9_ = 4.25, *p* < 0.001; TL: F_1,9_ = 2.20, *p* = 0.02). Indeed, conventional fed fish show generally higher weight and length than organic ones Figure 1 and Figure 2. Moreover, in both groups, fish exhibited a clear seasonal trend with an interruption of growth during cold months and a recovery of growth from May to the end of the sampling period (Tukey’s tests: March significantly differed from the following months, all *p* < 0.05. Figure 1 and Figure 2). The interaction between factors (diet x period) was evident at the end of the feeding period, when conventional fishes showed a higher increase in weight and TL than organic ones. Indeed, at the end of the trial weight and TL of conventional fishes significantly differed from those of organic ones (Tukey’s test: weight, *p* < 0.001; TL, *p* = 0.038).

**Figure 3:**
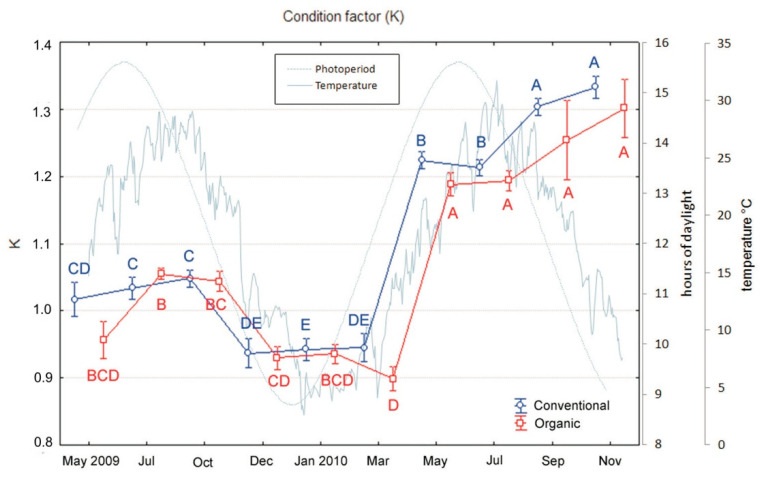
Variations in condition factor (K) of sea bass over an 18-month period (mean ± SE) reared under conventional and organic aquaculture. Different letters indicate significant differences in samplings within the same feeding system (organic or conventional; *p* < 0.05).

Regarding the condition factor “K”, the analyses evidenced a significant effect of both the groups and the sampling period (diet: F_1,9_ = 5.44, *p* = 0.02; period: F_1,9_ = 84.58, *p* < 0.001), but no interaction between the two factors (sampling × period). Indeed, in both groups, as observed for growth and TL, fish exhibited a seasonal trend in K values that were lower during cold months Figure 3 and significantly increased from May to the end of the feeding period (Tukey’s tests: March significantly differed from the following months, all *p* < 0.001. Figure 3). However, this trend was similar for conventional and organic fed fishes and no difference between the two groups was observed at the end of the feeding period.

### 3.2. Immunohistochemistry

**Figure 4:**
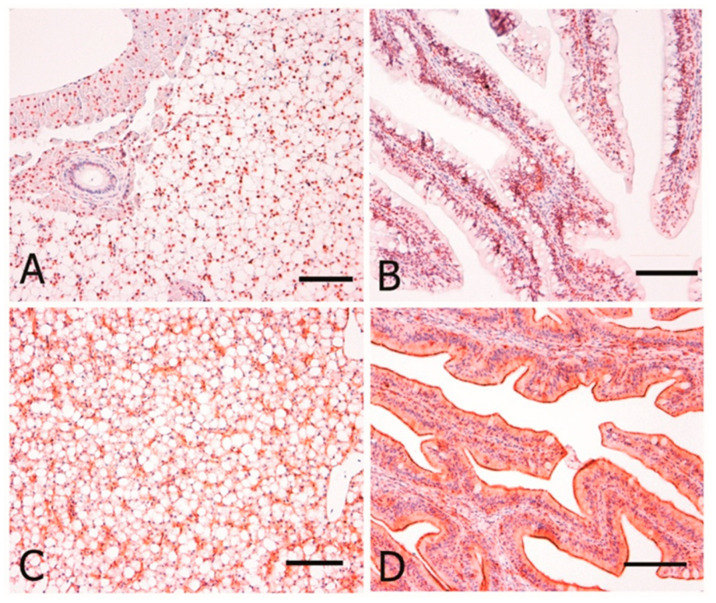
Immunohistochemical localization of CYP1A and 8-OHdG in sea bass. All panels are counterstained with Harryr’s hematoxylin. Scale bar: 50 µm. (**A**) Liver, strong and diffuse nuclear immunoreactivity of hepatocytes to anti-8-OHDG antibody; (**B**) Gut, nuclear immunoreactivity of most enterocytes to anti-8-OHDG antibody; (**C**) Liver, endothelial, and sinusoidal immunoreactivity of hepatocytes to anti-CYP-1A; (**D**) Gut, diffuse strong immunoreactivity of the intestinal mucosa to anti-CYP-1A.

**Figure 5:**
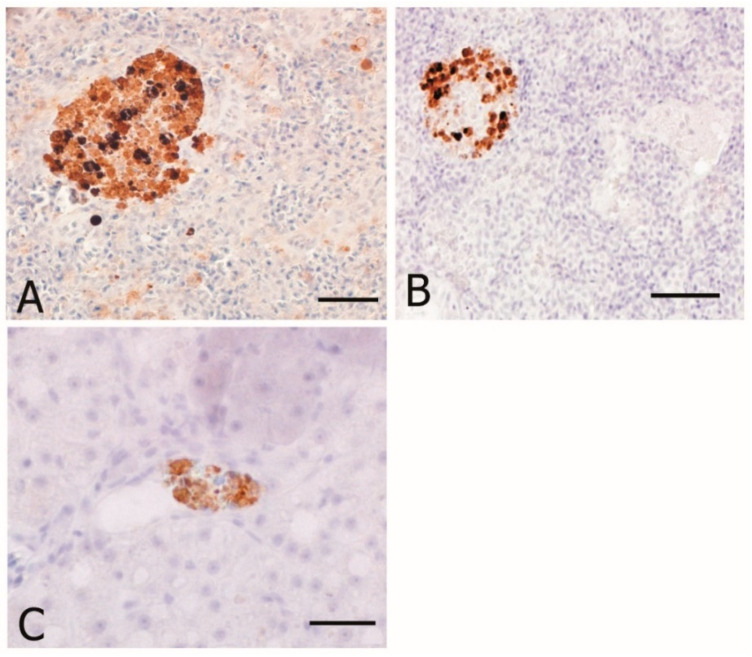
Immunohistochemical localization of HNE in sea bass. All panels are counterstained with Harryr’s hematoxylin. Scale bar: 50 µm. HNE-immunostaining is present in several melanomacrophage centers located in the parenchyma of (**A**) spleen; (**B**) kidney; (**C**) liver.

8-OHdG and HNE immunostaining were evaluated as DNA damage and oxidative stress markers, respectively, whereas CYP1A as environmental biomarker. Counting stained nuclei for 8-OHdG and CYP1A performed in liver and gut did not show any statistically difference between the two groups (ANOVA, p > 0.05; Figure 4). The anti-HNE staining was detected in the spleen, head kidney, and liver mostly in the MMCs and spare macrophages Figure 5. Immunopositivity was found both in organic and conventional samples with no differences in intensity between the two groups.

### 3.3. MMCs Count

**Figure 6:**
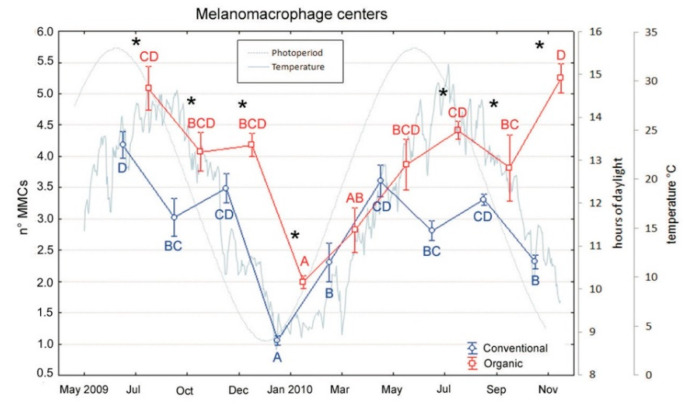
Variations in melanomacrophage centers number of sea bass over an 18-month period (mean ± SE) reared under conventional and organic aquaculture. Different letters indicate significant differences in samplings within the same feeding system (organic or conventional; *p* < 0.05). Asterisks indicate significant differences between feeding systems within the same sampling (*p* < 0.05).

MMCs counts, as markers of pollutant exposure, were performed on spleen sections and revealed several differences between the two groups Figure 6. In most samples, organic fed fishes exhibited a higher number of MMCs respect to conventional ones, except for March and May of the second year, where no significant differences were found (Tukey’s test, *p* < 0.01).

### 3.4. Total Glutathione (GSH)

**Figure 7:**
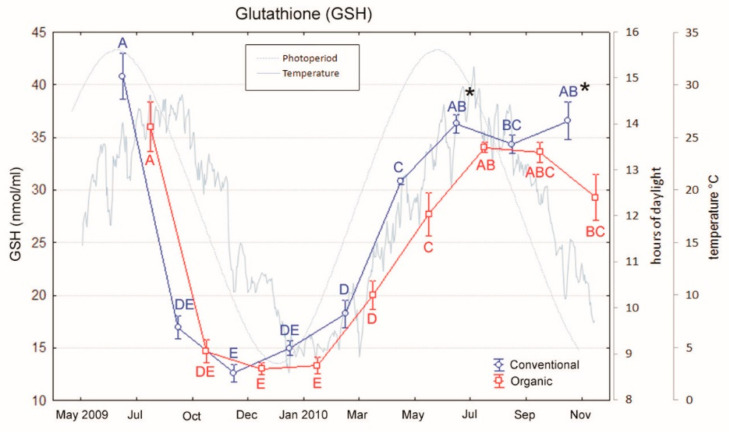
Variations in glutathione of sea bass over an 18-month period (mean ± SE) reared under conventional and organic aquaculture. Different letters indicate significant differences in samplings within the same feeding system (organic or conventional; *p* < 0.05). Asterisks indicate significant differences between systems within the same sampling (*p* < 0.05).

Total glutathione (GSH) was evaluated as oxidative stress marker. Total GSH assay showed significant differences between the two groups only in July and November of the second year (Tukey’s tests, p < 0.05; Figure 7). In both samplings, conventional fed fishes showed higher values than organic ones (35.9 ± 0.9 vs. 34.0 ± 1.8 and 36.2 ± 0.5 vs. 29.0 ± 2.2 nmol/mL).

### 3.5. IGF-I and IGF-II expression

Due to their role in growth regulation, IGF-I and IGF-II expression has been assessed. Neither the feeding condition nor the sampling period had a significant effect on IGF-I and IGF-II expression, and no significant interaction between the two factors was observed (IGF-I: diet: F_1,3_ = 0.13, *p* = 0.72; period: F_1,3_ = 0.27, *p* = 0.85; diet × period: F_1,3_ = 0.20, *p* = 0.89. IGF-II: diet: F_1,3_ = 0.004, *p* = 0.95; period: F_1,3_ = 0.78, *p* = 0.51; diet × period: F_1,3_ = 0.30, *p* = 0.82).

### 3.6. Proximate Composition and FA Analysis

The chemical composition of the experimental diets fed during the last period of growth of sea bass differed among treatments. In detail, the conventional diet showed lower ether extracts and ash compared to the organic one (17.2% vs. 20.3% and 7.73% vs. 12.3%, respectively), whereas an opposite trend was recorded for crude protein (44.5% vs. 40.7%) Table 1.

The proximate composition and fatty acid profile of sea bass fillets are reported in Table 2. Despite the starvation period prior to slaughtering, the ether extract content tended to be higher in sea bass that were fed with the diet containing the highest ether extract (*p* = 0.06), i.e., the conventional diet, in the last period of feeding compared to those fed the organic diet. Some differences among treatments were found in the fatty acid profile of sea bass. In fact, MUFAs content was higher in fillets of sea bass fed organic diet than conventional one (37.4% vs. 33.8%; *p* = 0.02), especially due to the higher content of C20:1n-9 and C22:1n-11. Besides, the PUFAs n-6 content was lower in the organic than in conventional fillets (12.0% vs. 15.8%; *p* = 0.04) due to the lower content of C18: 2n-6 (11.0% vs. 14.3%; *p* = 0.04).

## 4. Discussion

The present study investigated the productivity, the oxidative stress status and contaminant response in European sea bass fed an organic feed vs. a conventional one. As regards growth, conventional fed fishes showed a significant increase in growth at the end of the trial, suggesting that conventional feeding leads to a greater productivity. Nevertheless, the expression of IGF-I and IGF-II mRNA was similar throughout the whole experimental period confirming their roles on growth. As expected, the feed intake was affected by temperature, since both groups of fish experienced reduction of food intake during the cold season recording a temporary growth arrest [50]. In a different study on the effects of feed restriction in sea bass, Escobar-Aguirre et al. observed a decrease in plasma IGF-I levels of treated fish [51], thus confirming that the lower levels found in colder months could be related to the stop of feed intake due to the winter starvation period.

The diet composition did not seem to affect neither the oxidative stress parameters (GSH, 8-OHdG, HNE) nor the CYP1A expression, although several studies demonstrated the anti-oxidative effect of lipid and vitamin-rich diets [25,26]. It is likely that a more pronounced variation in the composition of such nutrients would need to affect antioxidant defenses. Nevertheless, total GSH exhibited some seasonal effects: Lowest values were found during cold months (October–March), whereas higher levels were seen during summer months. This seasonal tendency is in agreement with previous studies on the impact of temperature on fish GSH levels in various organs [52,53]. Moreover, it is well known that antioxidant levels are modified by food availability as Pascual et al. pointed out in their study in which they observed an increase of antioxidant activities and a disruption of GSH redox status in gilthead sea bream (*Sparus aurata*) kept under a food deprivation condition [54]. In this work, immunopositivity for HNE was detected in spleen, kidney, and liver, but with no differences in intensity between organic and conventionally fed fish. However, Fiocchi et al. in their study, aimed to evaluate the stress oxidative biomarkers in sea bass, also observed immunoprecipitates for HNE in the same organs [55], validating the use of this substance as stress biomarker.

Conversely, a significant higher expression of MMCs was observed in organic vs. conventional diet fed fish. However, this preliminary result, to be confirmed by a greater number of data, is discordant with Montero et al. [56] that reported an increased number of MMCs in *Sparus aurata* juveniles fed with a diet, low in EPA and DHA but could be related to a more efficient system of detoxification in the organic fishes or to the source of proteins and lipids within the organic diet. Organic farming condition could have developed a more reactive system of detoxification in fishes e.g., a higher MMCs rate leading to a better ability to respond versus potential stressors. Since few studies on MMCs centers in organic farmed sea bass are available, this result could be compared with what Magrone et al. [57] and Arciuli et al. [58] found in sea bass fed a diet enriched with polyphenols extracted from red grape. In fact, they also observed an increase of splenic and kidney MMCs number, area and activity in fish fed polyphenols, demonstrating how alternative feeding could indeed enhance the action of the immune and oxidative system. Otherwise, piscivorous fish represent an important accumulator of heavy metals and/or other pollutants since fish meal and oil used in organic aqua-feed are notoriously a source of contaminants [27]. As reported in salmon feeds [59,60], the fish oils obtained from feral pelagic fish species are considered the main source of persistent organic pollutants (POPs). In order to contain this problem, decontamination techniques have recently been developed to effectively remove persistent organic contaminants from fish oils [61,62]. Moreover, wild foods are notoriously highly nutritious but restricted in quantity and the use of fish-derived meal and oil, if on the one hand is closer to the natural life of the fish, on the other hand it does not match the principle of sustainability, causing overexploitation of fisheries and wild stocks [63] and should be reduced.

The chemical composition of the organic and conventional diets administered to the sea bass differed in terms of ether extract values, lower in the organic diet, and ash content and crude protein content higher compared with the conventional diet. The fillet FA profile is known to be strictly dependent on the diet. Indeed, the level of n-3 PUFAs largely depends on the dietary level and the types of supplemented fish meal and oils, especially vegetable oil [2,64,65,66]. Even if the precise source of fish oil used in the two diets was not declared by the producer, the high level of eicosenoic acid and cetoleic acid makes to presuppose that the organic diet was likely supplemented with a blend of fish oils containing herring oil. The organic aquaculture regulation fixes a maximum inclusion level of vegetable feeds at 60% of the diet and indirectly imposes a 40% level of fish meal and oil as a minimum and this should be reflected in the FA fillet composition of organic vs. conventional fed fishes as previously found by Trocino et al. [47].

## 5. Conclusions

In conclusion, this was a field study aimed to evaluate the growth performances as well as the expression of IGF-I and IGF-II mRNA, the oxidative stress and contaminant response in European sea bass fed with organic diet. The study was carried out as a pilot study in a small commercial plant to verify the feasibility of the production of sea bass using organic feed. These productive conditions impeded replications but give first results, although preliminary, on commercial production volumes. Our results highlighted a greater productivity in conventional fed fish comparing to the organic ones. The higher productivity was likely due to diet composition, since differences were significantly mitigated during starvation period. On the other hand, the considered oxidative stress and contaminant markers did not show any significant differences among groups. Feeding fish with diets with a content in fatty acids closer to that of wild fish definitely affect the nutritional value of the flesh in terms of the fatty acid profile. The consumption of the derived flesh could be considered more appropriate and healthier than the conventional one but due to the exploitation of some fisheries, wild-caught fish could not be the answer to the organic feed requirements. The findings and limitations raised in this study could stimulate a challenging debate in the field of organic fish nutrition in order to think a new concept of organic fish nutrition and consequently to better address required future researches in the same field.

## Figures and Tables

**Table 1:** Proximate composition (% as-fed) and fatty acid profile (% total fatty acids methyl esters) of the diets.

Rearing System (R)	Organic	Conventional
*Proximate composition*		
Water (%)	7.10	6.32
Ether extract (%)	20.3	17.2
Crude protein (%)	40.7	44.5
Crude fibre (%)	0.73	1.18
Ash (%)	12.3	7.73
*Fatty acid profile*		
C14:0	5.03	3.05
C15:0	0.37	0.23
C16:0	13.1	12.3
C17:0	0.35	0.32
C18:0	2.50	3.82
C20:0	0.21	0.28
Other SFAs	0.77	0.53
Total SFAs	22.4	20.6
C16:1n-7	3.90	3.03
C18:1n-7	3.93	3.99
C18:1n-9	18.5	21.3
C20:1n-9	2.76	0.84
C22:1n-9	0.71	0.13
C22:1n-11	8.08	0.62
C24:1n-9	0.46	0.19
Other MUFAs	0.81	0.35
Total MUFAs	39.0	30.4
C18:3n-3	2.92	4.96
C18:4n-3	5.82	0.79
C20:5n-3	4.47	5.96
C22:5n-3	0.91	0.74
C22:6n-3	5.90	3.10
PUFAs n-3	20.0	15.6
C18: 2n-6	13.3	29.3
C20:4n-6	0.24	0.31
PUFAs n-6	13.5	29.6
Ratio of n-3 to n-6 PUFAs	1.48	0.53
Other PUFAs	1.00	0.67
Total PUFAs	34.5	45.8
Unknown FAs	4.15	3.21

FAs: fatty acids; SFAs: Saturated FAs; MUFAs: monounsaturated FAs; PUFAs: polyunsaturated FAs.

**Table 2:** Proximate composition (% as-fed) and fatty acid profile (% of total fatty acid methyl esters) of sea bass fillets.

Rearing System (R)	Organic	Conventional	Probability	RSD
Live weight (g)	432	473	0.18	57
*Proximate composition*				
Water (%)	71.3	69.3	0.08	0.60
Ether extract (%)	7.93	9.72	0.06	0.47
Crude protein (%)	19.5	19.5	0.92	0.37
Ash (%)	1.14	1.16	0.41	0.01
*Fatty acid profile*				
C14:0	4.32	3.30	0.10	0.34
C15:0	0.53	0.40	0.15	0.06
C16:0	16.6	17.1	0.66	0.94
C17:0	0.54	0.49	0.32	0.04
C18:0	7.45	7.00	0.84	2.10
Other SFAs	4.18	2.90	0.22	0.72
Total SFAs	33.6	31.2	0.62	4.16
C16:1n-7	4.50	4.52	0.59	0.03
C18:1n-9	19.6	22.8	0.05	0.77
C18:1n-7	2.24	2.44	0.04	0.04
C20:1n-9	4.24	1.75	< 0.001	6.15
C22:1n-11	3.41	0.69	< 0.001	7.40
Other MUFAs	3.41	1.54	0.03	0.34
Total MUFAs	37.4	33.8	0.02	0.53
C18:3n-3	1.71	1.77	0.75	0.00
C18:4n-3	1.04	0.73	0.09	0.10
C20:5n-3	2.72	4.41	0.10	0.56
C22:5n-3	0.70	0.96	0.29	0.18
C22:6n-3	3.36	3.67	0.79	1.06
PUFAs n-3	10.1	12.0	0.45	2.10
C18: 2n-6	11.0	14.3	0.04	0.69
C20:4n-6	0.34	0.46	0.08	0.04
PUFAs n-6	12.0	15.8	0.04	0.79
Ratio of n-3 to n-6 PUFAs	0.83	0.76	0.64	0.12
Other PUFAs	3.03	3.63	0.10	0.20
Total PUFAs	23.8	29.9	0.18	2.96
Unknown FAs	5.14	5.13	0.99	0.00

FAs: fatty acids; SFAs: Saturated FAs; MUFAs: monounsaturated FAs; PUFAs: polyunsaturated FAs.
